# Comparative analysis of the lateral and posterolateral trajectories for fixation of the sacroiliac joint—a cadaveric study

**DOI:** 10.1186/s13018-020-02013-w

**Published:** 2020-10-22

**Authors:** Christopher Payne, Stephen Jaffee, Isaac Swink, Daniel Cook, Matthew Yeager, Michael Oh, Gary Schmidt, Derek P. Lindsey, Scott A. Yerby, Boyle Cheng

**Affiliations:** 1 Neuroscience Institute, Allegheny General Hospital, Pittsburgh, PA USA; 2 Department of Neurosurgery, University of California Irvine, Irvine, CA USA; 3 Orthopaedic Institute, Allegheny General Hospital, Pittsburgh, PA USA; 4 SI-BONE, Inc., 471 El Camino Real, Suite 101, Santa Clara, CA 95051 USA

**Keywords:** Lateral approach, Posterolateral approach, Sacroiliac joint, Sacroiliac fusion, Virtual trajectory, Bone mineral density

## Abstract

**Background:**

A number of minimally invasive sacroiliac (SI) joint fusion solutions for placing implants exist, with reduced post-operative pain and improved outcomes compared to open procedures. The objective of this study was to compare two MIS SI joint fusion approaches that place implants directly across the joint by comparing the ilium and sacrum bone characteristics and SI joint separation along the implant trajectories.

**Methods:**

Nine cadaveric specimens (*n* = 9) were CT scanned and the left and right ilium and sacrum were segmented. The bone density, bone volume fraction, and SI joint gap distance were calculated along lateral and posterolateral trajectories and compared using analysis of variance between the two orientations.

**Results:**

Iliac bone density, indicated by the mean Hounsfield Unit, was significantly greater for each lateral trajectory compared to posterolateral. The volume of cortical bone in the ilium was greater for the middle lateral trajectory compared to all others and for the top and bottom lateral trajectories compared to both posterolateral trajectories. Cortical density was greater in the ilium for all lateral trajectories compared to posterolateral. The bone fraction was significantly greater in all lateral trajectories compared to posterolateral in the ilium. No differences in cortical volume, cortical density, or cancellous density were found between trajectories in the sacrum. The ilium was significantly greater in density compared with the sacrum when compared irrespective of trajectory (*p* < 0.001). The posterolateral trajectories had a significantly larger SI joint gap than the lateral trajectories (*p* < 0.001).

**Conclusion:**

Use of the lateral approach for minimally invasive SI fusion allows the implant to interact with bone across a significantly smaller joint space. This interaction with increased cortical bone volume and density may afford better fixation with a lower risk of pull-out or implant loosening when compared to the posterolateral approach.

## Background

Sacroiliac joint (SIJ) fusion surgery has evolved over the last several decades, with early procedures requiring extensive open muscular dissections and bone grafting [1]. Conditions that could lead to degenerative sacroiliitis and/or SIJ disruption (requiring SIF fusion) may include asymmetric distribution of force across the joint—caused by leg length discrepancies or gait abnormalities, persistent joint strain over time, scoliosis, pregnancy, and lumbar or lumbosacral fusion—caused by increases in angular motion and average stress across the SIJ [2–5]. Currently, a number of minimally invasive solutions for placing SIJ implants exist, with research suggesting MIS solutions are associated with reduced post-operative pain and better peri-operative outcomes compared to open procedures [6, 7].

Although SIJ fusion success rates are reported at 80-85% and fusion rates between 35 and 100%, bone quality continues to be a concern when instrumenting the spine [8–11]. Therefore, researchers and surgeons continue to search for means of better fixation to avoid long term failure. Specifically, patients that display lower bone density or severe osteoporotic characteristics may exhibit qualities that contraindicate this procedure due to lack of sufficient bone density and quality for proper fixation. Additionally, the quality of bone found in the sacrum has proved difficult for use in fusion surgeries because of its lack of cortical bone and lower density [12, 13]. Thus, this study will compare the overall bone, cortical, and cancellous bone density, and bone fraction lying along the two most common MIS SIJ fusion trajectories that place implants directly across the joint, the posterolateral and lateral approaches, by using a simulated surgical model (other dorsal approaches that place implants within the joint were not considered in this analysis). This will be accomplished through the introduction of virtual cylindrical dowels across the joint and the analysis of the different qualities of bone in the lateral versus posterolateral trajectories.

The lateral approach is seen as an attractive method of surgical entry because it is thought to be less invasive, as surgeons do not need to transect large quantities of soft tissue including ligaments and tendons. This minimally invasive procedure begins with a small incision on the lateral buttock in order to reach the ilium, with implants placed across the SI joint traversing the ilium into the sacrum [14]. The posterolateral approach was developed because it was the more direct trajectory of the two and also did not necessitate retracting through large quantities of soft tissue dissection, with implants placed starting near the PSIS, traversing the ilium, crossing the ligamentous portion of the SI joint, and into the sacral ala [15]. While these differences are apparent in the clinical literature, our goal was to ascertain the differences in the trajectories not based in their directness of approach or soft tissue characteristics, but the overall quality of the bone that was traversed in patients with conditions such as osteoporosis and low bone density.

It is important to note that any fixation method (including methods utilizing surgical screws or press-fit implants) are dependent upon bone quality—evaluated with metrics such as bone mineral density and ratio of cancellous to cortical bone. There may be substantial ramifications to fixation if the patient suffers from low bone density, such as in osteoporotic patients. It is well understood that patients with this condition typically lose significant amounts of cancellous bone, while their cortical bone remains respectively intact [16]. The diminished volume of cancellous bone may significantly impact both short- and long-term fixation, potentially causing pseudoarthrosis at the SIJ [17]. Thus, the implementation of a more heavily emphasized cortical bone approach may provide preferential implant anchoring, in spite of the eventual waning of cancellous bone.

Using computer topography (CT) segmentation, computer modeling, and Hounsfield units (HU)—as a proxy for bone density and characteristics, we intend to characterize and compare the abovementioned metrics to gain further insight into the nature of the SIJ and potentially provide evidence for superior characteristics in one trajectory over the other.

## Methods

### Imaging

Dual-energy X-ray absorptiometry (DEXA) scanning was obtained on the L4 vertebrae on each specimen using a clinical DEXA scanner (Discovery Wi, Hologic). Specimens were then CT scanned (Somatom, Siemens, Munich, Germany) with a consistent imaging protocol (slice thickness = 0.6 mm, KVP = 140). The voxels contained within the bony volume of the sacrum and the left and right ilium were segmented to form models of the 3D surface of each body identified through segmentation, using commercially available software (ScanIP, Synopsys, Mountain View, CA). Five of these CT scans were performed with phantoms of known density corresponding to cancellous and cortical bone.

### Region of interest (ROI) determination

**Fig. 1:**
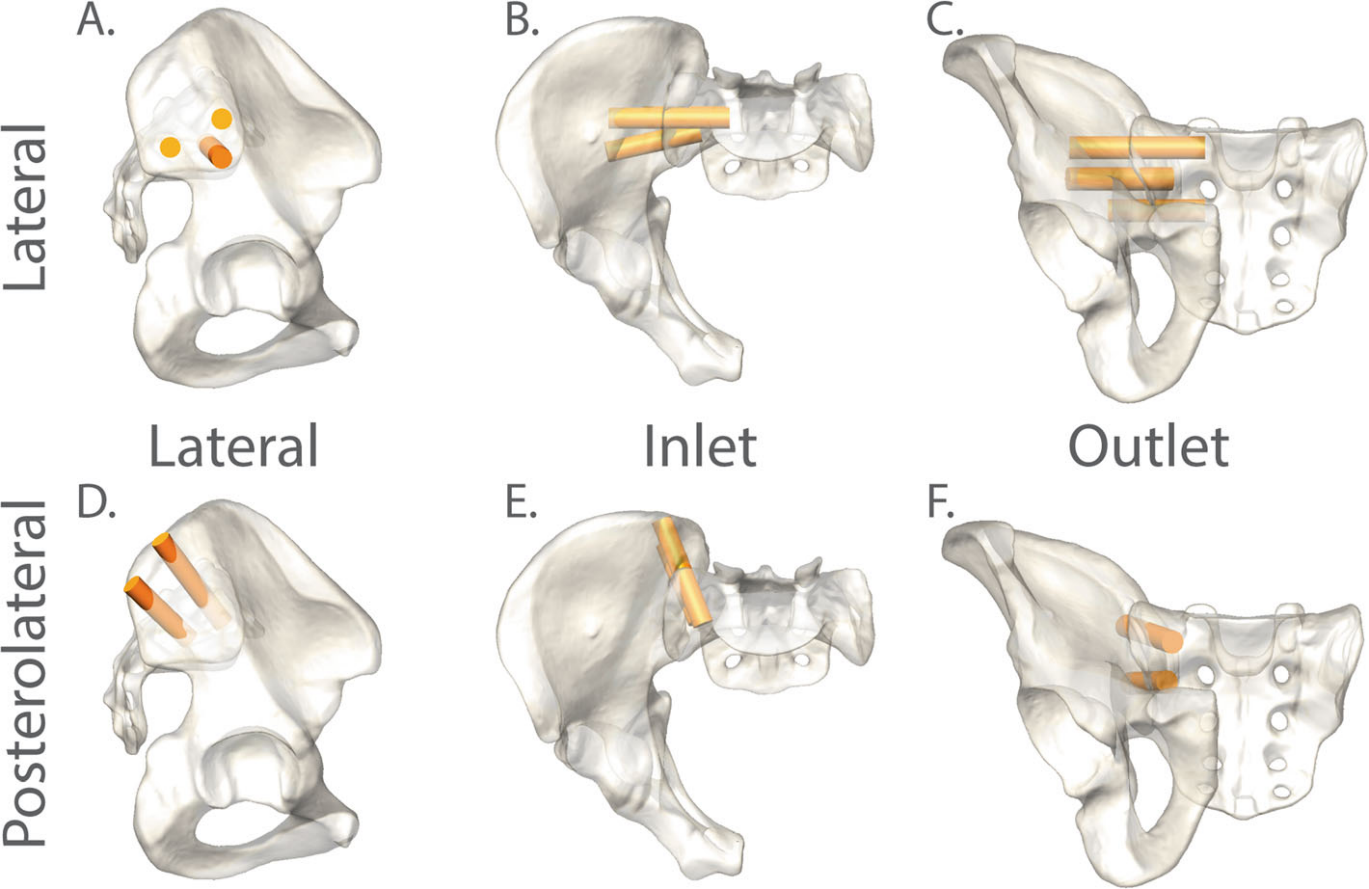
Illustration of the virtual dowels used to restrict analysis for each trajectory

The trajectories for each of three lateral implants (top, middle, bottom) and two posterolateral implants (top or “PL1”, bottom or “PL2”) were established by selecting two landmarks for each implant based on the technique guides of commercially available implants (Table 1). Landmarks were selected based on the endpoint of each trajectory—corresponding to the projected distal tip of each implant from a surgeon’s perspective—and a second point lying in the trajectory path on the proximal side of the ilium. OsiriX (Pixmeo SARL, Geneva, Switzerland) was used to place all landmark fiducials. Using the coordinates of the fiducial markers chosen for each specimen, a line was created to serve as the axis of virtual cylinders 12 mm in diameter (Fig. 1). The volume within these virtual cylinders follows the implant trajectory and represents the bone that would directly interact with implants. As such, data from within this ROI was used for analysis and trajectory comparison.

**Table 1:** Start and end points for the 3 lateral and 2 posterolateral implant trajectories

**Lateral orientation**		
**Trajectory**	**Start point**	**End point**
Top implant	Lateral to the middle of the first sacral body, distal to the alar line, following the slope of the ala	12 o’clock position above S1 foramina
Middle implant	Lateral to the S2 foramen, between the first and second sacral body, angled approximately 15-20° ventral-to-dorsal from horizontal	1 cm in advance of the S1 foramen
Bottom implant	Lateral to the middle of the second sacral body, ensure the implant is roughly parallel with the top and middle implants in an outlet view	Between the S1 and S2 foramen near the lateral border of the S1 foramen
**Posterolateral orientation**		
**Trajectory**	**Start point**	**End point**
Top implant (PL1)	At the lateral aspect of the PSIS. Follow the trajectory that is 10-15° lateral-to-medial and 0-10° cranial-to-caudal	At the ala of the sacrum about 1 cm from the anterior sacral cortex
Bottom implant (PL2)	2-3 cm posterior of the sacral ala, in line with the S2 pedicle, follow a similar trajectory to the top implant	In the ala of the sacrum about 1 cm from the anterior sacral cortex

### Calculation methodology

The voxel readings (HU), within each of ROI, were the subjects of analysis. Cancellous bone was defined as the interval of voxels with a density between ≥ 219 and < 867, while cortical bone corresponds to any voxels ≥ 867. Voxel readings within the ROI were averaged and analyzed in three separate groups: (1) the entire ROI (A_T_), (2) cortical bone (A_C_), and (3) cancellous bone (A_Ca_) to characterize the bone quality. In addition, the number of voxels falling within the predetermined density intervals was recorded to determine the volume of both types of bone. Bone fraction (BF) was defined as the fraction of cortical or cancellous bone out of the total ROI volume. Joint gap represents the distance between the bony surfaces on either side of the SIJ, which corresponds to the medial aspect of the ilium and lateral aspect of the sacrum. These surfaces were delineated based on segmentation of cortical bone, and the joint gap mapping was calculated by measuring the closest distance between the joint surfaces, which intersects with the virtual trajectory of each implant.

### Statistical testing

A repeated-measures ANOVA was conducted on joint gap distance (measured in mm) by trajectory (top, middle, bottom, PL1, and PL2) with Bonferroni-corrected post hoc analysis. A 2-way multivariate analysis of variance on mean HU, cortical density, cancellous density, cortical volume, and bone fraction was conducted over the independent variables: body (sacrum/ilium) and trajectory, and Bonferroni-corrected post hoc analysis for pairwise comparisons between trajectories within each body was also computed.

## Results

**Fig. 2:**
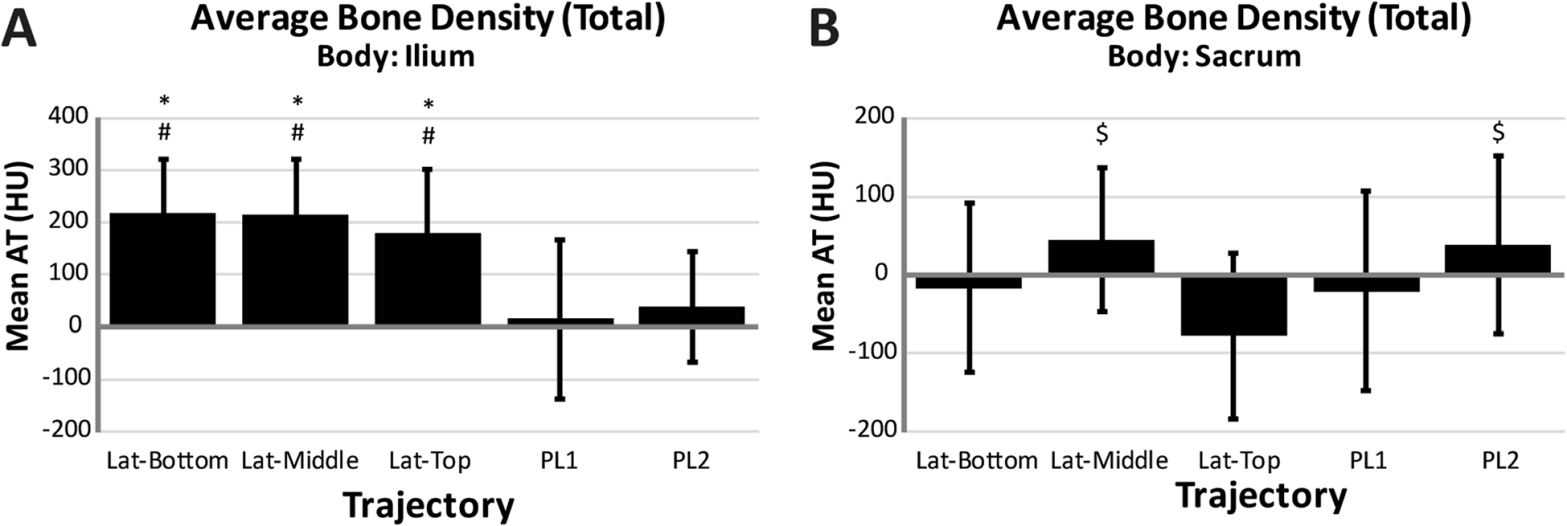
(**a**) Average density associated with the ilium portion of each virtual dowel; asterisk indicates significant difference compared to PL1; number sign indicates significant difference compared to PL2, (**b**) average density associated with the sacrum portion of each virtual dowel; dollar sign indicates significant difference compared to Lat-Top

**Fig. 3:**
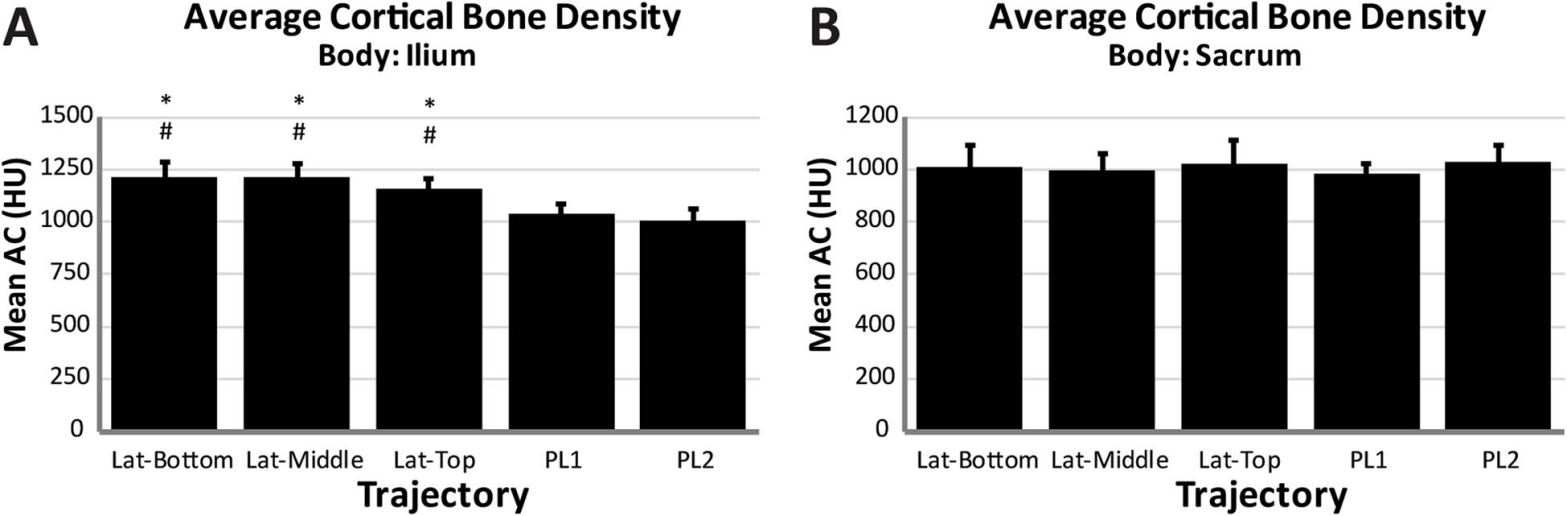
(**a**) Average cortical bone (HU ≥ 867) density measured within the ilium for each virtual dowel; asterisk indicates significant difference compared to PL1; number sign indicates significant difference compared to PL2, (**b**) average cortical bone (HU ≥ 867) density measured within sacrum for each virtual dowel

**Fig. 4:**
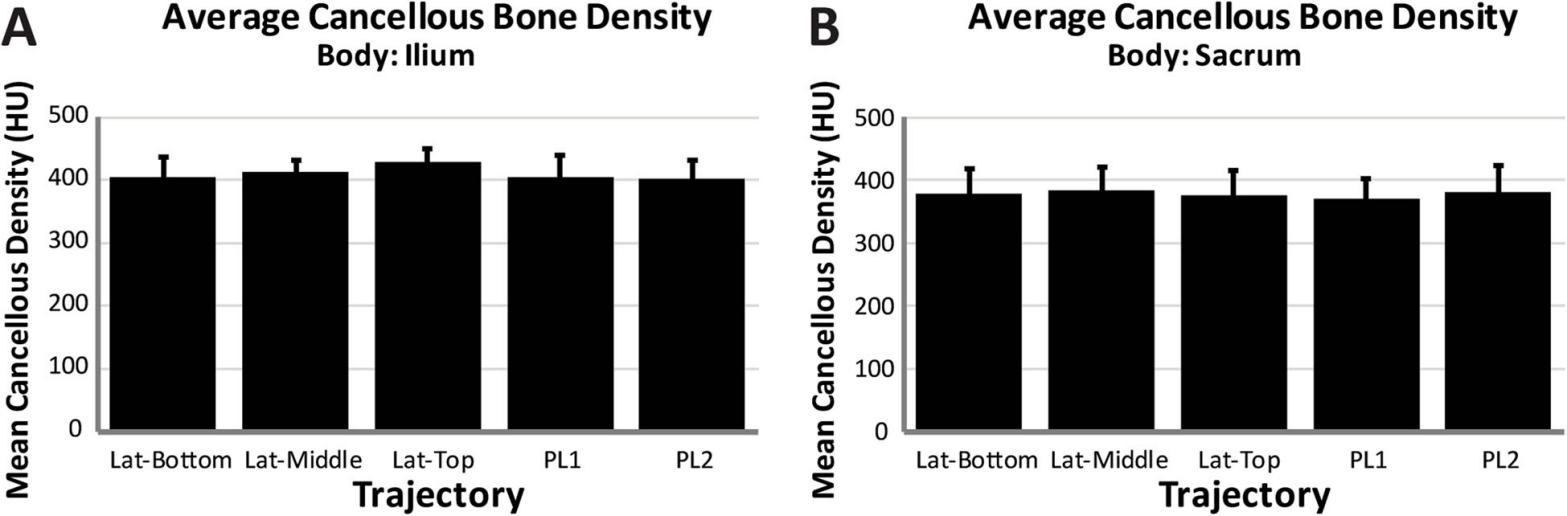
(**a**) Average cancellous bone (867 > HU ≥ 219) density measured within the ilium for each virtual dowel, (**b**) average cancellous bone (867 > HU ≥ 219) density measured within the sacrum for each virtual dowel

**Fig. 5:**
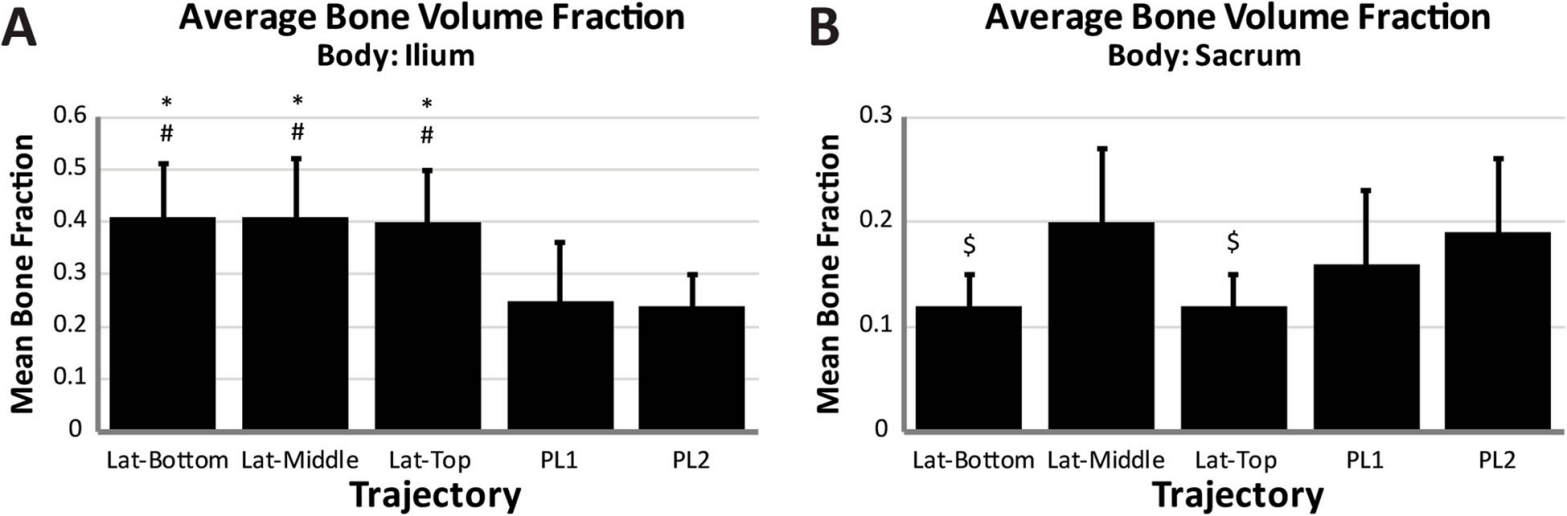
(**a**) Average bone volume fraction measured within the ilium for each trajectory; asterisk indicates significant difference compared to PL1; number sign indicates significant difference compared to PL2, (**a**, **b**) average bone volume fraction measured within the sacrum for each trajectory; dollar sign indicates significant difference compared to Lat-Middle; number sign indicates significant difference compared to PL2

Nine specimens (mean age 60 years, range 24-80; 6 female, 3 male) were used and the average specimen L4 vertebrae bone mineral density was 0.948 g/cm^2^ (SD = 0.194) (Table 2). Based on the individual L4 T-scores, two specimens were normal, four specimens had osteopenia, and three had osteoporosis. Utilizing phantoms of known density, the mean ilium *A*_Ca_ was 219 HU (*n* = 5, SD = 3), and the mean ilium A_C_ was 867 HU (*n* = 5, SD = 9). The mean ilium *A*_T_ of the three lateral trajectories was significantly greater than both posterolateral trajectories (*p* ≤ 0.004, *p* ≤ 0.002 respectively), with no differences observed between the lateral trajectories (Fig. 2). In the sacrum, the top trajectory showed significantly lower mean *A*_T_ compared to both middle (*p* = 0.026) and PL2 (*p* = 0.048). The mean cortical volume in the ilium was significantly greater in the middle trajectory compared to all others (*p* ≤ 0.015) and was significantly greater in the top and bottom compared to both PL1 and PL2 (*p* < 0.001) (Table 3). No differences in mean cortical volume were observed between trajectories within the sacrum. The mean cortical density in the ilium was significantly greater for all lateral trajectories compared to posterolateral trajectories (*p* < 0.001) (Fig. 3). No significant differences were observed within the sacrum between any of the trajectories. There were no significant differences in cancellous density between the different trajectories in either the ilium or sacrum (Fig. 4). The mean BF in the ilium was significantly greater in all lateral trajectories compared to posterolateral (*p* < 0.001) (Fig. 5). In the sacrum, the mean bone fraction was significantly greater for middle compared to both top (*p* = 0.035) and bottom (*p* = 0.033). Results showed the number of voxels identified as cortical and cancellous bone, bone fraction within each trajectory, and the gap distance of PL1 were significantly greater than all other trajectories (*p* < 0.001). The same results applied to PL2 were also significantly greater than top (*p* = 0.003), middle (*p* < 0.001), and bottom (*p* = 0.005) trajectories (Table 3).

**Table 2:** Specimen demographics

Specimen number	Age	Sex	Height (in)	Weight (lbs)	L4 BMD (g/cm2)	T-score
1	73	M	71	280	0.722	−3.8
2	75	M	71	350	1.098	−0.4
3	63	F	68	230	0.939	−1.6
4	64	M	67	270	1.001	−2.3
5	80	F	65	160	1.347	2.1
6	70	F	61	250	0.986	−1.2
7	39	F	70	129	0.738	−3.4
8	55	F	67	135	0.879	−2.2
9	24	F	56	110	0.825	−2.6
Avg.	60		66	213	0.948	−1.71
SD	18		5	83	0.194	1.77

**Table 3:** Summary voxel count, bone fraction, and gap distance for each trajectory (mean ± standard deviation)

	Ilium				Sacrum				Joint gap distance (mm)
Trajectory	Cortical voxels	Cancellous voxels	Total voxels	Bone fraction	Cortical voxels	Cancellous voxels	Total voxels	Bone fraction	
PL1	296 ± 393	2926 ± 1301	12415 ± 1843	0.25 ± 0.11	23 ± 39	923 ± 401	5838 ± 1165	0.16 ± 0.07	6.53 ± 0.69
PL2	109 ± 138	2436 ± 774	10778 ± 2713	0.24 ± 0.06	53 ± 62	1276 ± 418	7165 ± 1725	0.19 ± 0.07	3.79 ± 0.49
Top	767 ± 340	2314 ± 485	7801 ± 1554	0.40 ± 0.10	103 ± 129	1818 ± 545	15415 ± 1805	0.12 ± 0.03	1.50 ± 0.29
Middle	977 ± 278	3075 ± 809	10072 ± 1212	0.41 ± 0.11	44 ± 52	1132 ± 401	5998 ± 1360	0.20 ± 0.07	1.21 ± 0.21
Bottom	766 ± 353	2512 ± 753	8042 ± 1260	0.41 ± 0.10	44 ± 54	1069 ± 283	9040 ± 1761	0.12 ± 0.03	1.63 ± 0.31

See Table 1 for trajectory descriptions

**Fig. 6:**
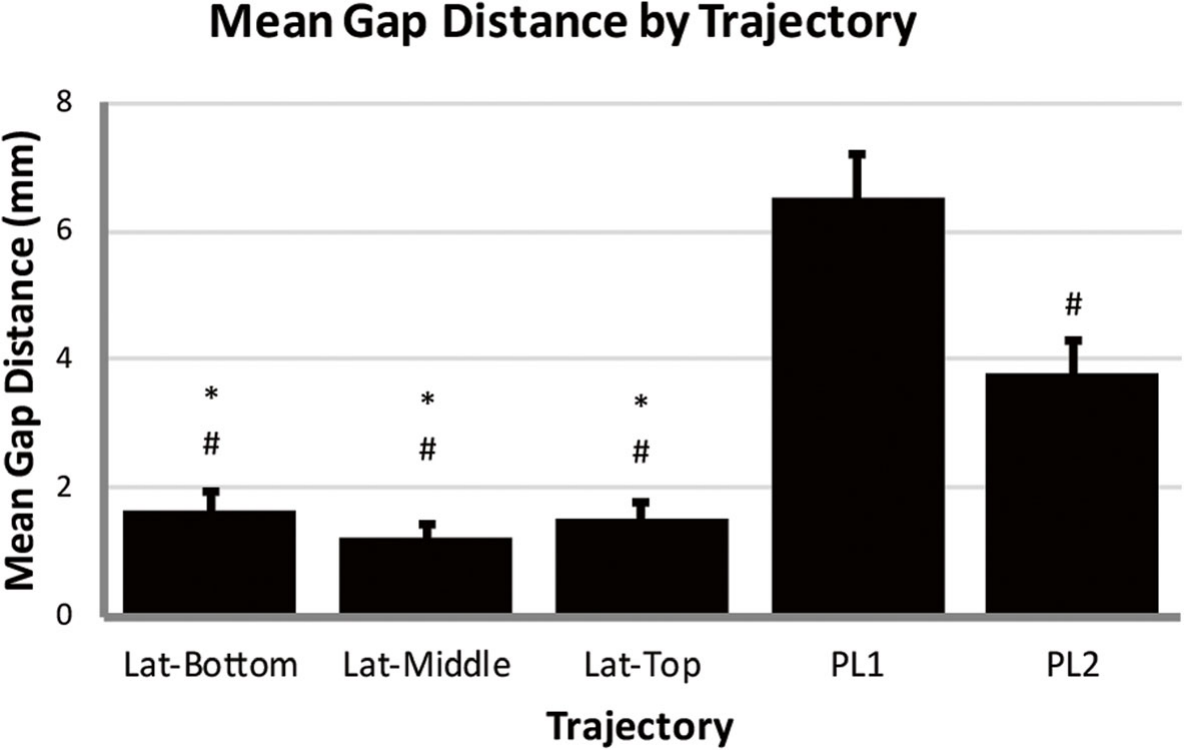
Average joint gap distance spanned by each virtual dowel; number sign indicates significant difference compared to PostLat1; asterisk indicates a significant difference compared to PostLat2

Joint gap distances from all trajectories can be found in Table 3. All three lateral trajectories were shown to have a significantly smaller joint gap distance than the posterolateral trajectories (Fig. 6). No significant differences were found in distance between any of the trajectories in the lateral orientation, but the superior posterolateral trajectory was found to result in a significantly larger gap distance than the inferior posterolateral trajectory.

## Discussion

Through the comparison of virtual CT scan trajectories using cadaveric models, we demonstrated that there were significant differences between the characteristics of bone within the lateral and posterolateral SIJ fusion approaches. Specifically, we showed that the cortical volume in the ilium was significantly greater in the lateral compared to the posterolateral approach. Both approaches utilize the ilium and sacrum for fusion; therefore, the characteristics of the bone within each trajectory are critical to understanding the relationship between the location of the implant and the quality of the bone therein. We found that sacral bone is not as dense or as high in cortical volume as that found in the ilium (Table 3). This finding is consistent with work performed by McLauchlan and Gardner, who studied a number of adult cadaveric specimens, showing that iliac cancellous bone volume was significantly greater than that of sacral cancellous bone [18]. Moreover, the volume of cortical bone in the ilium ranges from 2 to 20 times that in the sacrum for corresponding trajectories (Table 3), which may be explained only in part by the fact that the trajectories traverse two cortices in the ilium and only one in the sacrum—indicating a significantly greater cortical thickness in the ilium compared to the sacrum.

This study also shows that fixation within the ilium may be greater with the lateral approach than the posterolateral approach and that no significant difference in fixation is likely to be achieved when looking at the device from a sacral standpoint. However, the bone fraction encountered along the lateral approach through the ilium was consistently shown to be of higher density and include more cortical bone. While the clinical results from this finding are unknown, DeCoster et al. tested two commercially available bone screws on a synthetic bone model, consisting of urethane foam, and showed that as the density of this model increased, the pullout force also increased in a linear fashion, thus demonstrating the correlation between higher density fixation and increased pullout force [19]. It may be hypothesized that the increase in cortical bone through the lateral approach may provide a similar increase in pullout force, which may translate to preferable fixation and fewer complications such as implant loosening. Furthermore, Halvorsen et al. demonstrated similar results on cadaveric bone with pullout strength, which was shown to increase in a linear fashion in relation to bone mineral density, providing further evidence that the use of a trajectory with significantly higher bone density means a potential for increased fixation [20]. Moreover, Santoni et al. assessed the bone density across two pedicle screw trajectories in the lumbar spine using a quantitative CT scanner and determined that (1) their novel trajectory included higher bone density and (2) it showed that this higher density leads to a higher pullout force in mechanical testing—similar to our research [21]. This study leans on the use of a CT model and the use of volumetric regions of interest in the lumbar spine, which may be generalized to the SIJ for the purposes of correlating bone density to pullout force. While our study did not incorporate this additional modality of testing, the biomechanics literature provides evidence that higher bone density does correspond to increased pullout force. A number of cadaveric biomechanical studies utilizing lateral or posterolateral implants have been performed to investigate the influence on sacroiliac joint stability [22–24], but a direct comparison using the two approaches may provide an interesting follow-up to this analysis. Based on these findings, we hypothesize that a lateral approach may provide a fixation that is more secure and less likely to loosen when compared to a SIJ fusion utilizing the posterolateral approach.

Additionally, previous work by Bruna-Russo et al. also demonstrated that better fixation was accomplished with an implant orientation that was farther and more parallel (i.e., lateral) relative to the center of SIJ rotation which lends further credence to the benefits of the lateral trajectory as opposed to the posterolateral [25]. Although it was not the primary focus of the current study, the lateral approach allows for longer implants to be placed in the denser cancellous bone of the sacral vertebral body and may further reduce implant loosening [12, 13, 26–28].

Analysis of the joint gap distance along each trajectory indicates significantly greater distance between opposing bone surfaces for the posterolateral orientation compared to that of the lateral trajectory—which may present a comparatively large impediment to fusion across the joint. The mean joint gap distance for the lateral trajectories was 1.46 mm while that of the posterolateral was 5.16 mm. The results of this analysis for the lateral trajectories are reinforced by the work of McLauchlan and Gardner who demonstrated mean cartilage thickness of the sacral and iliac joint surfaces to be 1.81 and 0.8 mm respectively [18]. These findings indicate direct contact of the cartilaginous surfaces traversed by the lateral trajectories analyzed within the current study. In addition, Bruna-Rosso et al. demonstrated that implant trajectories that damage the interosseous ligament resulted in less stability of a treated SIJ [25]. Further clinical study is warranted to definitively assess the relative merits of fusion across the joint along these two orientations, with regard to rate and success.

One limitation of this study was the lack of use of known density phantoms during each of the CT scans of our specimens. The specimens were not all scanned with cortical and cancellous phantoms due to logistical reasons, which prevented us from directly converting HU to mass density for each specimen scanned. However, given the relatively low variance in the HU values for the cortical and cancellous phantoms scanned, and the fact that all specimens were scanned on the same machine using the same protocol, we are confident that the HU ranges used for defining cortical and cancellous bone are appropriate. Furthermore, because the calculated relative densities were taken from different locations in the same specimens, it is unlikely that there is a bias in outcomes between the trajectories measured due to variance in CT scanning parameters between specimens. The results found within this study are consistent with outcomes of lateral fixation compared with posterolateral fixation as described above. The current study focused on two trajectories that place implants directly across the SI joint for stabilization; as such, results for dorsal approaches that place implants within the joint (intra-articular) remain an area for future research. Finally, the present study was facilitated by industry grant support, which may be interpreted as a potential source of bias.

## Conclusion

The lateral SIJ approach includes higher density bone, more cortical bone, higher bone fraction, and smaller SIJ gap when compared to the posterolateral approach. The lateral approach may lead to greater fixation of implants in the ilium because of a significantly greater bone density. This study provides a significant model for future studies about patient-specific trajectories based on our metrics of interest. It will be imperative to increase the knowledge of this joint and the significance of these findings in a biomechanical assessment using both the computer-modeled trajectories and then biomechanical stability testing to ascertain if the results directly correlate to one another.

## Data Availability

All data generated or analyzed during this study are included in this published article.

## References

[1] Stark JG, Fuentes JA, Fuentes TI, Idemmili C (2011). The history of sacroiliac joint arthrodesis: a critical review and introduction of a new technique. Curr Orthop Pract..

[2] Ha K-Y, Lee J-S, Kim K-W (2008). Degeneration of sacroiliac joint after instrumented lumbar or lumbosacral fusion: a prospective cohort study over five-year follow-up. Spine..

[3] DePalma MJ, Ketchum JM, Saullo TR (2011). Etiology of chronic low back pain in patients having undergone lumbar fusion. Pain Med..

[4] Ivanov AA, Kiapour A, Ebraheim NA, Goel V (2009). Lumbar fusion leads to increases in angular motion and stress across sacroiliac joint: a finite element study. Spine..

[5] Liliang P-C, Lu K, Liang C-L, Tsai Y-D, Wang K-W, Chen H-J (2011). Sacroiliac joint pain after lumbar and lumbosacral fusion: findings using dual sacroiliac joint blocks. Pain Med..

[6] Graham Smith A, Capobianco R, Cher D, Rudolf L, Sachs D, Gundanna M, Kleiner J, Mody MG, Shamie AN (2013). Open versus minimally invasive sacroiliac joint fusion: a multi-center comparison of perioperative measures and clinical outcomes. Ann Surg Innov Res..

[7] Martin CT, Haase L, Lender PA, Polly DW (2020). Minimally invasive sacroiliac joint fusion: the current evidence. Int J Spine Surg..

[8] Duhon BS, Bitan F, Lockstadt H, Kovalsky D, Cher D, Hillen T. Triangular titanium implants for minimally invasive sacroiliac joint fusion: 2-year follow-up from a prospective multicenter trial. Int J Spine Surg. 2016;10:Article 13. 10.14444/3013 PMC4852595 27162715

[9] Polly DW, Swofford J, Whang PG, Frank C, Glaser JC, Limoni RP, Cher DJ, Wine KD, Sembrano JN, INSITE Study Group. Two-year outcomes from a randomized controlled trial of minimally invasive sacroiliac joint fusion vs. non-surgical management for sacroiliac joint dysfunction. Int J Spine Surg. 2016 Aug 23;10:Article 28. 10.14444/3028 PMC5027818 27652199

[10] Sturesson B, Kools D, Pflugmacher R, Gasbarrini A, Prestamburgo D, Dengler J (2017). Six-month outcomes from a randomized controlled trial of minimally invasive SI joint fusion with triangular titanium implants vs. conservative management. Eur Spine J..

[11] Yoshihara H (2012). Sacroiliac joint pain after lumbar/lumbosacral fusion: current knowledge. Eur Spine J..

[12] Hoel RJ, Ledonio CGT, Takahashi T, Polly DW. Sacral bone mineral density (BMD) assessment using opportunistic CT scans: sacral bone mineral density. J Orthop Res [Internet]. 2016 Jul [cited 2016 Jul 11]; Available from: http://doi.wiley.com/10.1002/jor.23362. 10.1002/jor.23362 27391403

[13] Richards AM, Coleman NW, Knight TA, Belkoff SM, Mears SC (2010). Bone density and cortical thickness in normal, osteopenic, and osteoporotic sacra. J Osteoporos..

[14] Geisler F. Stabilization of the sacroiliac joint with the SI-bone surgical technique. Neurosurg Focus. 2013 Jul;35(2 Suppl):Video 8. 10.3171/2013.V2.FOCUS13195 23829857

[15] Beck CE, Jacobson S, Thomasson E (2015). A retrospective outcomes study of 20 sacroiliac joint fusion patients. Cureus..

[16] Osterhoff G, Morgan EF, Shefelbine SJ, Karim L, McNamara LM, Augat P (2016). Bone mechanical properties and changes with osteoporosis. Injury..

[17] Fischer CR, Hanson G, Eller M, Lehman RA (2016). A systematic review of treatment strategies for degenerative lumbar spine fusion surgery in patients with osteoporosis. Geriatr Orthop Surg Rehabil..

[18] McLauchlan GJ, Gardner DL (2002). Sacral and iliac articular cartilage thickness and cellularity: relationship to subchondral bone end-plate thickness and cancellous bone density. Rheumatol Oxf Engl..

[19] DeCoster TA, Heetderks DB, Downey DJ, Ferries JS, Jones W (1990). Optimizing bone screw pullout force. J Orthop Trauma..

[20] Halvorson TL, Kelley LA, Thomas KA, Whitecloud TS, Cook SD (1994). Effects of bone mineral density on pedicle screw fixation. Spine..

[21] Santoni BG, Hynes RA, McGilvray KC, Rodriguez-Canessa G, Lyons AS, Henson M (2009). a. W, Womack WJ, Puttlitz CM. Cortical bone trajectory for lumbar pedicle screws. Spine J Off J North Am Spine Soc..

[22] Dubé-Cyr R, Aubin C-É, Villemure I, Bianco R-J, Godio-Raboutet Y, Arnoux P-J (2020). Biomechanical analysis of two insertion sites for the fixation of the sacroiliac joint via an oblique lateral approach. Clin Biomech Bristol Avon..

[23] Soriano-Baron H, Lindsey DP, Rodriguez-Martinez N, Reyes PM, Newcomb A, Yerby SA, Crawford NR (2015). The effect of implant placement on sacroiliac joint range of motion: posterior vs trans-articular. Spine..

[24] Lindsey DP, Parrish R, Gundanna M, Leasure J, Yerby SA, Kondrashov D (2018). Biomechanics of unilateral and bilateral sacroiliac joint stabilization: laboratory investigation. J Neurosurg Spine..

[25] Bruna-Rosso C, Arnoux P-J, Bianco R-J, Godio-Raboutet Y, Fradet L, Aubin C-É. Finite element analysis of sacroiliac joint fixation under compression loads. Int J Spine Surg. 2016;10:Article 16. 10.14444/3016 PMC4943166 27441174

[26] Peretz AM, Hipp JA, Heggeness MH (1998). The internal bony architecture of the sacrum. Spine..

[27] Zheng Y, Lu WW, Zhu Q, Qin L, Zhong S, Leong JCY (2000). Variation in bone mineral density of the sacrum in young adults and its significance for sacral fixation. Spine..

[28] Schicho A, Gebhard F, Richter P (2016). CT-based bone density assessment for iliosacral screw trajectories. J Orthop Allied Sci..

